# Fear of sleep in first responders: associations with trauma types, psychopathology, and sleep disturbances

**DOI:** 10.1093/sleepadvances/zpad053

**Published:** 2023-12-01

**Authors:** Anthony N Reffi, David A Kalmbach, Philip Cheng, Peter Tappenden, Jennifer Valentine, Christopher L Drake, Wilfred R Pigeon, Scott M Pickett, Michelle M Lilly

**Affiliations:** Thomas Roth Sleep Disorders and Research Center, Henry Ford Health, Detroit, MI, USA; Thomas Roth Sleep Disorders and Research Center, Henry Ford Health, Detroit, MI, USA; Thomas Roth Sleep Disorders and Research Center, Henry Ford Health, Detroit, MI, USA; Department of Psychology, Northern Illinois University, DeKalb, IL, USA; Department of Psychology, Northern Illinois University, DeKalb, IL, USA; Thomas Roth Sleep Disorders and Research Center, Henry Ford Health, Detroit, MI, USA; U.S. Department of Veterans Affairs, VISN 2 Center of Excellence for Suicide Prevention, Canandaigua, NY, USA; Department of Psychiatry, University of Rochester Medical Center, Rochester, NY, USA; Center for Translational Behavioral Science, Florida State University College of Medicine, Tallahassee, FL, USA; Department of Psychology, Northern Illinois University, DeKalb, IL, USA

**Keywords:** trauma-induced insomnia, nocturnal vigilance, public safety, emergency responders, occupational trauma

## Abstract

**Study Objectives:**

Fear of sleep contributes to insomnia in some individuals with posttraumatic stress disorder (PTSD) but remains uncharacterized in first responders, a population with high rates of insomnia and PTSD. We evaluated the clinical relevance of fear of sleep in first responders by (1) examining its relationship with trauma types and clinical symptoms and (2) assessing differences in fear of sleep severity between those reporting provisional PTSD, insomnia, or both.

**Methods:**

A cross-sectional study of 242 first responders across the United States (59.2% male, 86.4% white, 56.2% law enforcement officers, 98.7% active duty, and *M*_years of service_ = 17). Participants completed the Fear of Sleep Inventory-Short Form and measures of trauma history, psychopathology (e.g. PTSD), and sleep disturbances (insomnia and trauma-related nightmares).

**Results:**

Fear of sleep was associated with trauma types characterized by interpersonal violence and victimization, as well as symptoms of PTSD, depression, anxiety, stress, alcohol use problems, insomnia, and trauma-related nightmares. Fear of sleep was most pronounced among first responders reporting provisional PTSD comorbid with insomnia compared to those with PTSD or insomnia only. Post hoc analyses revealed PTSD hyperarousal symptoms and trauma-related nightmares were independently associated with fear of sleep, even after adjusting for the remaining PTSD clusters, insomnia, sex, and years of service.

**Conclusions:**

Fear of sleep is a clinically relevant construct in first responders that is associated with a broad range of psychopathology symptoms and is most severe among those with cooccurring PTSD and insomnia. Fear of sleep may merit targeted treatment in first responders. This paper is part of the Sleep and Circadian Health in the Justice System Collection.

**Statement of Significance**

Emerging evidence indicates fear of sleep underpins insomnia among individuals with posttraumatic stress disorder (PTSD). First responders are routinely exposed to duty-related trauma and might be vulnerable to a fear of sleep, which could exacerbate their already high rates of insomnia and PTSD. Yet, no research has investigated fear of sleep in this population. We found fear of sleep is a clinically meaningful construct in first responders, especially those with comorbid PTSD and insomnia, which is associated with more severe depression, anxiety, stress, alcohol use problems, and trauma-related nightmares. Clinicians working with first responders may consider screening for sleep fears using the Fear of Sleep Inventory, as doing so might uncover important treatment targets to improve sleep quality and overall mental health.

Posttraumatic stress disorder (PTSD) is a debilitating condition marked by pronounced difficulties falling and staying asleep [1]. These insomnia symptoms affect upwards of 91% of individuals diagnosed with PTSD [2] and limit patients’ response to first-line treatments for the disorder [3–5]. Insomnia symptoms are also among the most common residual symptoms to persist even after the core PTSD symptoms are successfully treated [5–8] and predict worsening PTSD symptoms up to 9 months after PTSD treatment [9]. Taken together, it appears that factors apart from PTSD drive sleep disturbances in trauma-exposed populations that, if left unresolved, hinder PTSD treatments and heighten the risk of PTSD symptom relapse. Therefore, a better understanding of how insomnia symptoms are maintained in trauma-exposed populations may improve our ability to optimally treat PTSD.

Recent evidence supports a fear of sleep as one important mechanism in this relationship [10]. Fear of sleep is defined as a fear of being vulnerable and unsafe during sleep [10]. Fear of sleep is thought to develop in response to certain PTSD symptoms, including nightmares [11, 12], that engender fears of losing control, reexperiencing trauma through nightmares, or both [10]. These fears promote maladaptive safety behaviors (e.g. avoiding sleep, sleeping with the lights on) that temporarily assuage fear and anxiety but cause further long-term impairments in sleep. Thus, while fear of sleep might initially be *triggered* by PTSD symptoms, it could evolve into an independent condition *sustained* by safety behaviors that worsen insomnia, PTSD, and overall health and functioning [10, 13]. Indeed, fear of sleep is most severe in individuals with comorbid PTSD and insomnia [14, 15] and has been linked with more severe nightmares, anxiety, and depression [10]. One population impacted by PTSD and insomnia that may be at risk of fear of sleep is first responders.

First responders—including law enforcement officers, paramedics, and 9-1-1 (emergency) telecommunicators—routinely encounter duty-related trauma and occupational stressors (e.g. shift work) that increase their risk for psychopathology relative to the general population [16–18], including PTSD [19], insomnia [20], and nightmares [21]. Moreover, first responders experience disproportionate rates of interpersonal trauma types associated with a fear of sleep, namely sudden violent death, and sexual and physical assault [17, 22]. Consequently, first responders may be vulnerable to developing fear of sleep, which could worsen sleep quality, compound comorbid conditions, and interfere with their performance of critical occupational duties. Identifying fear of sleep as a clinically meaningful construct among first responders might inform early screening and intervention efforts to improve their sleep health and overall well-being, which could precipitate downstream positive effects on the communities they serve.

The goal of this cross-sectional study was to characterize and assess the clinical relevance of fear of sleep in first responders. Specifically, we aimed to replicate previous evidence that fear of sleep is (1a) associated with exposure to interpersonal trauma types and (1b) more severe clinical symptoms (e.g. PTSD, insomnia, nightmares), and (2) is more pronounced in first responders with comorbid PTSD and insomnia compared to those with PTSD only or insomnia only.

## Materials and Methods

We collected cross-sectional online survey data using Qualtrics from 242 first responders across the United States from mid-October 2021 through mid-November 2021. We recruited participants using professional contacts of the first and senior authors (AR and ML), first responder social media sites, and snowballing methods of these two recruitment strategies. We informed participants that the survey was voluntary and without compensation, and its purpose was to learn more about the sleep health of first responders. Participants who provided their consent online then completed the survey and were debriefed following survey completion. We collected anonymous data only (i.e. no personally identifying information). All procedures were approved by Northern Illinois University’s Institutional Review Board.

## Measures

### Demographics and occupational characteristics

Participants completed a survey to provide demographic and occupational information including sex, race and ethnicity, first responder occupation, years of service, work schedule (e.g. past month night shift work), relationship status, and a single item on bed sharing (“If you are living with a partner, how frequently do you sleep in the same bed at roughly the same time?”).

### Fear of Sleep Inventory-Short Form

The Fear of Sleep Inventory-Short Form (FoSI-SF) is a 13-item questionnaire that assesses fears in relation to sleep (e.g. “I was fearful of letting my guard down while sleeping”) and safety behaviors to cope with such fear (e.g. “I slept with a light on to feel safer”) [23]. Participants indicated how often they experienced sleep fears or engaged in associated safety behaviors over the past month using a 5-point scale ranging from 0 (not at all) to 4 (nearly every night), for a possible total score range of 0–52. We computed the full-scale sum score to use as our outcome variable, with greater scores indicating more severe fear of sleep. The Cronbach’s alpha for the FoSI-SF in the current study was 0.88.

### Life events checklist for DSM-5

The life events checklist (LEC-5) is a 17-item inventory of lifetime exposure to 16 potentially traumatic events, with item 17 assessing exposure to “any other stressful event or experience.” Participants indicated whether they experienced each event by indicating either that it happened to them personally, they witnessed it happen to someone else, they learned about it happening to a close family member or close friend, or they were exposed to it as part of their job. Consistent with the fifth edition of the *Diagnostic and Statistical Manual of Mental Disorders* (DSM-5) [24], we coded any of these responses as a positive exposure to any of the 16 potentially traumatic events (we excluded item 17 because of its ambiguity; see above). We then summed all positive trauma exposures to compute a “trauma load” variable, with greater scores indicating the total amount of traumatic life events experienced (cumulative lifetime trauma). Additionally, we computed three empirically derived clusters of trauma types previously shown to differentially correlate with mental health [25]: Accidental/injury traumas (e.g. transportation accident; the sum of LEC-5 items 1–4 and 12), victimization traumas (e.g. physical assault; the sum of LEC-5 items 6, 8, and 9), and predominant death threat traumas (containing mostly death-related traumas, e.g. assault with a weapon; the sum of LEC-5 items 5, 7, 10, 11, and 13–16).

### Posttraumatic stress disorder checklist for DSM-5

The posttraumatic stress disorder checklist (PCL-5) is a 20-item questionnaire that assesses DSM-5 PTSD symptoms along four clusters (intrusions, avoidance, negative alterations in cognitions and mood, and hyperarousal) [26]. Participants rated their PTSD severity over the past week in relation to a stressful life event using a 5-point scale ranging from 0 (not at all) to 4 (extremely), for a possible total score range of 0–80. We computed both a sum score, with greater scores indicating more severe PTSD symptoms, and a dichotomized variable using a cutoff score of ≥33 to group participants by provisional PTSD status [27]. The Cronbach’s alpha for the PCL-5 in the current study was 0.95.

### Depression, Anxiety, and Stress Scales

The Depression, Anxiety, and Stress Scales (DASS-21) [28] is a 21-item questionnaire comprised of three interrelated scales that assess symptoms of depression, anxiety, and stress (e.g. nervous tension, difficulty relaxing, and irritability). Participants reported these symptoms over the past week using a 4-point scale ranging from 0 (did not apply to me at all) to 3 (applied to me very much, or most of the time), for a possible total score range of 0–21 for each subscale. We summed each subscale and doubled the scores to render them comparable to those derived from the full 42-item version [28]. To measure clinically significant symptoms, we then dichotomized these variables using cutoff scores of ≥14 (depression), ≥10 (anxiety), and ≥19 (stress), all of which indicate at least moderate symptom severity [28]. The Cronbach’s alphas for the DASS-21 subscales were 0.90 (depression), 0.78 (anxiety), and 0.84 (stress).

### Alcohol Use Disorder Identification Test

The Alcohol Use Disorder Identification Test (AUDIT-10) is a 10-item questionnaire on alcohol consumption, with higher sum scores indicating increased alcohol-related problems, for a possible total score range of 0–40. We computed an ordinal variable to indicate risk for alcohol use disorder, with 0 = abstinent (sum score = 0), 1 = low-risk (sum score = 1–7), 2 = hazardous (sum score = 8–14), and 3 = moderate-severe alcohol use disorder (sum score ≥15) [29]. We also used a cutoff score of ≥8 to indicate hazardous or harmful drinking that leads to negative health consequences and increased risk for alcohol use disorder [30]. The Cronbach’s alpha for the AUDIT-10 in the current study was 0.83.

### Insomnia Severity Index

The Insomnia Severity Index is a 7-item questionnaire that assesses DSM-5 insomnia symptoms over the past 2 weeks [31]. Participants rated their insomnia severity using a four-point scale, for a possible total score range of 0–28. We computed both a sum score, with greater scores indicating more severe insomnia symptoms, and a dichotomized variable using a cutoff score of ≥15 to group participants by provisional insomnia disorder status [32]. The Cronbach’s alpha for the Insomnia Severity Index total score in the current study was 0.91.

### Trauma-related nightmare distress

We measured trauma-related nightmare distress using the PCL-5 item 2 (“Repeated, disturbing dreams of the stressful experience”). Participants indicated how much they were bothered by trauma-related nightmares over the past week using a five-point scale ranging from 0 (not at all) to 4 (extremely). We used both the item sum score, with greater scores indicating worse nightmare distress, and a dichotomized variable using a cutoff score of ≥2 (indicating at least moderate distress) to measure clinically significant nightmares [26].

## Data Analysis

We analyzed data using SPSS Version 26. We first performed Pearson’s bivariate correlations to assess the convergent validity of fear of sleep with trauma load (including for each trauma type cluster), mental health symptoms, alcohol use problems, and sleep disturbances. We also assessed exploratory associations between fear of sleep, first responder occupation, shift work status, sex, years of service, and bed sharing.

Next, we performed a two-way factorial analysis of covariance (ANCOVA) to examine the main effects of PTSD (with two levels: (1) no PTSD and (2) provisional PTSD [PCL ≥ 33]) and insomnia (with two levels: (1) no insomnia and (2) provisional insomnia [ISI ≥ 15]) on fear of sleep, and the interaction effect between PTSD and insomnia on fear of sleep (provisional PTSD × provisional insomnia). We assessed sex, years of service, and trauma load as potential covariates and retained each if *p* < .05. We followed up significant interaction effects with Bonferroni-adjusted simple-effects analysis. We report partial eta squared (*η*^2^) to indicate effect sizes, with values of 0.01 = small, 0.06 = medium, and 0.14 = large [33].

## Results

### Participant demographics, trauma exposure, and clinical symptoms

Most participants were male (59.2%), white (86.4%), and worked in law enforcement (56.2%). The second most common first responder occupation was 9-1-1 telecommunicator (31.8%). Almost all participants were on active duty (98.7% reported currently working at least part-time as a first responder), with an average of 17 years of service (*SD* = 10.16, range = 4 months–45 years). The full demographic and occupational data are reported in Table 1.

**Table 1.** Demographics and Occupational Characteristics (*N* = 242)

Variables	*n* (%)^1^	*M* ± *SD*
Sex (male)	132 (59.2%)	
Race^2^		
White	209 (86.4%)	
Black or African American	11 (4.5% )	
American Indian or Alaska Native	4 (1.7%)	
Asian American	3 (1.2%)	
Native Hawaiian or Pacific Islander	1 (0.4%)	
Other	10 (4.1%)	
Hispanic/Latino(a)	11 (4.9%)	
First responder occupation^2^		
Law enforcement officer	136 (56.2%)	
9-1-1 telecommunicator	77 (31.8%)	
Firefighter	17 (7%)	
EMS/EMT	8 (3.3%)	
Other (e.g. support staff)	5 (2.1%)	
Years of service		16.92 ± 10.16
Active duty	224 (98.7%)	
Veteran of the armed services	34 (15.1%)	
Length of assigned shift		
8 hours	93 (41.3%)	
10 hours	81 (36%)	
12 hours	42 (18.7%)	
≥12 hours	9 (4%)	
Number of times needed to stay at work past end of shift (past 2 months)		7.16 ± 5.37
Number of overtime shifts (past 2 months)		
Voluntary		4.46 ± 4.55
Mandatory		4.72 ± 5.52
Night shift work (past month)		
Yes	131 (58.2%)	
Sometimes (rotate)	20 (8.9%)	
Married	144 (63.7%)	
Partner also works as first responder	36 (20.2%)	
Works same shift as partner	64 (40.3%)	
Shares bed with partner	87 (49.2%)	

EMS/T, Emergency medical services/technician; Active duty = currently working at least part-time as a first responder; Shift work = regularly worked hours outside of 07:00–18:00 over the past month; Shares bed with partner = always or almost always sleeps in the same bed as a partner at the same time (6–7 days per week).

^1^Pertentages account for missing data.

^2^Participants could select multiple responses.

There were high rates of lifetime exposure to traumatic events as indexed by the LEC-5 (*M*_number of events_ = 12.48, Median = 13, *SD* = 2.82, range = 2 – 16)^1^ (Table 2). The most reported trauma type cluster was predominant death threat traumas (*M*_number of events_ = 5.37, *SD* = 1.69), followed by accidental/injury (*M*_number of events_ = 4.54, *SD* = 0.87) and victimization trauma types (*M*_number of events_ = 2.57, *SD* = 0.84). Approximately one-quarter to one-third of our sample reported clinically significant levels of psychopathology: PTSD (23.1%), depression (28.1%), anxiety (35.5%), and stress (25.2%). Eighteen percent of our sample reported hazardous or harmful alcohol use, and 5.4% scored positive for moderate-severe alcohol use disorder. Regarding sleep, most of our sample reported trauma-related nightmare distress over the past week (53.4%), with 23.2% indicating clinically significant distress. Over one-third of participants screened positive for provisional insomnia (37.8%). See Table 2 for rates of trauma exposure (grouped by trauma type clusters), clinically significant mental health symptoms, hazardous alcohol use, and clinically significant sleep disturbances.

**Table 2.** Rates of Lifetime Trauma Exposure, Clinically Significant Mental Health Symptoms, Hazardous Alcohol Use, and Sleep Disturbances (*N* = 242)

Variable	*n* (%)^1^	*M* ± *SD*
Accidental/injury traumas		4.54 ± 0.87
Natural disaster	211 (87.6%)	
Fire or explosion	221 (91.7%)	
Transportation accident	237 (98.3%)	
Serious accident at work, home, or during recreational activity	220 (91.3%)	
Life-threatening illness or injury	205 (85.1%)	
Victimization traumas		2.57 ± 0.84
Physical assault (e.g. being hit, beaten up)	226 (93.8%)	
Sexual assault (including attempted rape)	204 (84.6%)	
Other unwanted or uncomfortable sexual experiences	189 (78.4%)	
Predominant death threat traumas		5.37 ± 1.69
Exposure to toxic substances (e.g. dangerous chemicals)	139 (57.7%)	
Combat or exposure to a war zone	111 (46.1%)	
Captivity (e.g. being kidnapped or held hostage)	97 (40.2%)	
Assault with a weapon (e.g. being shot, stabbed)	216 (89.6%)	
Severe human suffering	185 (76.8%)	
Sudden violent death (e.g. homicide, suicide)	229 (95.0%)	
Sudden accidental death	227 (94.2%)	
Serious injury, harm, or death you caused to someone else	91 (37.8%)	
Posttraumatic stress disorder (PTSD)	56 (23.1%)	
Depression^2^	68 (28.1%)	
Anxiety^2^	86 (35.5%)	
Stress^2^	61 (25.2%)	
Hazardous or harmful alcohol consumption	41 (18.0%)	
Trauma-related nightmares	56 (23.2%)	
Insomnia	88 (37.8%)	

Participants could select multiple traumas. Participants indicated traumatic exposure through either direct personal experience, witnessing it happen to someone else, learning about it happening to a close family member or close friend, or being exposed to it as part of their job.

PTSD, posttraumatic stress disorder scale for DSM-5 (PCL-5) sum score ≥33, indicating provisional PTSD over the past week; depression, anxiety, and stress = Depression Anxiety and Stress Scale (DASS-21) sum scores ≥14, 10, or 19, respectively, indicating at least moderate severity for each symptom subscale over the past week; hazardous or harmful alcohol consumption = Alcohol Use Disorders Identification Test sum score ≥8; trauma-related nightmares = clinically significant distress over trauma-related nightmares assessed for the past week using the PCL-5 item 2 (“Repeated, disturbing dreams of the stressful experience”) score ≥2 (moderate distress); insomnia = Insomnia Severity Index sum score ≥15, indicating provisional insomnia disorder over the past 2 weeks.

^1^Pertentages account for missing data.

^2^Subscale scores were doubled to render them comparable to full DASS-42 scale

### Correlations between fear of sleep, trauma types, and clinical symptoms

Fear of sleep had a small and positive correlation with trauma load (*r* = 0.18, *p* = .008). Of the specific trauma types, fear of sleep correlated most with greater exposure to predominant death threat traumas (*r* = 0.18, *p* = .007) followed by victimization trauma types (*r* = 0.16, *p* = .015); fear of sleep was not significantly correlated with experiencing accidental/injury traumas (*r* = 0.06, *p* = .345). As displayed in Table 3, fear of sleep was weakly correlated with greater alcohol use problems (*r* = 0.15, *p* = .023), and moderately to strongly associated with more severe symptoms of PTSD, depression, anxiety, stress, insomnia, and trauma-related nightmares (*r* range = 0.44–0.52, *p*s < .01). We also found large differences in mean levels of fear of sleep between first responders with clinically significant mental health symptoms and sleep disturbances compared to those with relatively milder symptoms (Supplementary Table S1). Exploratory analyses showed no differences in fear of sleep between law enforcement officers and 9-1-1 telecommunicators, nor between those who regularly worked night shifts over the past month as compared to those who worked standard schedules that occurred during 07:00–18:00 hours (Supplementary Table S1). Lastly, exploratory correlations showed fear of sleep had a small to moderate relationship with female sex (*r* = 0.18, *p* = .008), fewer years of service (*r* = −0.20, *p* = .003), and more nights of sleeping alone in bed (*r* = −0.25, *p* = .001).

**Table 3.** Descriptive Statistics and Bivariate Correlations Among Study Variables (*N* = 242)

Scale	1	2	3	4	5	6	7	8	9	10	11	12	13	14	15
1.Fear of sleep	—														
2.Sex	.18**	—													
3.Years of service	−.20**	−.34**	—												
4.Bed sharing	−.25**	−.20**	.15	—											
5.Trauma load	.18**	.01	.08	.08	—										
6.Accidental/injury traumas	.06	.03	.13	.11	.76**	—									
7.Victimization traumas	.16*	.15*	−.05	.04	.75**	.47**	—								
8.Death threat traumas	.18**	−.08	.09	.06	.91**	.52**	.51**	—							
9.PTSD severity	.52**	.08	−.14*	−.17*	.17**	.07	.15*	.18**	—						
10.Depression severity	.46**	.08	−.15*	−.27**	.02	−.08	.04	.05	.71**	—					
11.Anxiety severity	.48**	.15*	−.09	−.22**	.09	.04	.16*	.05	.60**	.60**	—				
12.Stress severity	.45**	.07	−.05	−.22**	.11	.01	.11	.13*	.70**	.68**	.67**	—			
13.Alcohol use	.15*	−.06	−.07	.13	−.01	−.04	.03	−.01	.08	.11	.20**	.16*	—		
14.Trauma-related nightmares	.44**	.11	−.11	−.09	.13	.02	.11	.15*	.67**	.43**	.39**	.38**	0.10	—	
15.Insomnia severity	.47**	.11	−.11	−.26**	.16*	.04	.15*	.17**	.60**	.51**	.47**	.60**	0.07	0.37**	—
*n*	228	223	225	117	241	241	241	241	242	242	242	242	228	242	233
Min	0	1	0.33	0	2	1	0	0	0	0	0	0	0	0	0
Max	32	2	45	4	16	5	3	8	68	42	32	42	3	4	28
Mean	3.53	1.41	16.92	2.71	12.48	4.54	2.57	5.37	20.89	9.80	7.70	14.84	1.13	0.87	12.28
Median	1.00	1.00	17.00	3.00	13.00	5.00	3.00	6.00	18.00	8.00	6.00	14.00	1.00	1.00	12.00
*SD*	5.89	0.49	10.16	1.45	2.82	0.87	0.84	1.69	15.75	8.38	6.51	7.86	.66	1.01	6.57

Pearson and point-biserial correlations.

Fear of sleep = Fear of Sleep Inventory-Short Form (FoSI-SF) sum score; sex (1 = male, 2 = female); years of service = years of working in public safety; bed sharing = frequency of sleeping in same bed as partner at roughly the same time (0 = never or almost never, 1 = rarely [1–2 days per week], 2 = about half the time [3–4 days per week], 3 = most of the time [5–6 days per week], 4 = almost always [6–7 days per week]); trauma load = life events checklist for DSM-5 (LEC-5) sum score, indicating total number of traumatic life events experienced (“happened to me,” “witnessed it,” “learned about it,” or “part of my job”); accidental/injury traumas = sum of LEC-5 items 1–4 and 12; victimization traumas = sum of LEC-5 items 6, 8, 9; predominant death threat traumas = sum of LEC-5 items 5, 7, 10, 11, 13–16; PTSD severity = PTSD checklist for DSM-5 sum score evaluated for the past week; depression, anxiety, and stress severity = Depression, Anxiety, and Stress Scales (DASS-21) subscales sum scores evaluated for the past week (doubled to be comparable to the DASS-42); alcohol use = alcohol consumption scored by risk levels using the Alcohol Use Disorders Identification Test (AUDIT-10) (0 = abstinent [AUDIT-10 score = 0], 1 = low-risk [AUDIT-10 score = 1–7], 2 = hazardous [AUDIT-10 score = 8–14], and 3 = moderate-severe alcohol use disorder [AUDIT-10 score ≥ 15]); trauma-related nightmares = distress over trauma-related nightmares assessed for the past week using the PCL-5 item 2 (“Repeated, disturbing dreams of the stressful experience”; 0 = not at all, 1 = a little bit, 2 = moderately, 3 = quite a bit, 4 = extremely); insomnia severity = Insomnia Severity Index sum score evaluated for the past 2 weeks.

* *p* < .05; ** *p* < .01

### Differences in fear of sleep by PTSD, insomnia, and comorbid PTSD-insomnia

**Figure 1.**
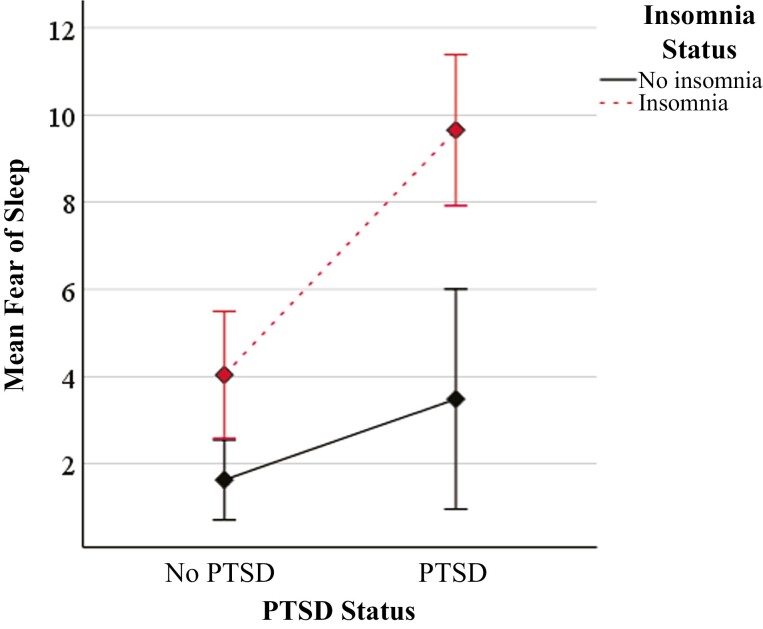
Fear of sleep is most pronounced in first responders with comorbid PTSD and insomnia (*N* = 242). Note. Two-way ANCOVA results display a significant interaction between PTSD and insomnia status on the severity of fear of sleep (*F*(1, 213) = 4.49, *p* = .035, *η*^2^ = 0.02). PTSD, posttraumatic stress disorder checklist for DSM-5 sum score ≥33 evaluated for the past week; insomnia = Insomnia Severity Index sum score ≥15 evaluated for the past 2 weeks; mean fear of sleep = Fear of Sleep Inventory-Short Form mean score (range = 0–52) evaluated for the past month. Sex and years of service are included as covariates (not pictured). Error bars represent 95% confidence intervals.

Sex and years of service were significant covariates in our ANCOVA and were retained in the model, whereas each trauma load variable was nonsignificant and thus excluded from further analysis. While adjusting for sex and years of service, there were significant main effects for both PTSD (*F*(1, 213) = 17.51, *p* < .001, *η*^2^ = 0.08) and insomnia on fear of sleep (*F*(1, 213) = 23.04, *p* < .001, *η*^2^ = 0.10), indicating a medium and medium-to-large effect size, respectively. There was also a significant interaction between PTSD and insomnia status on the severity of fear of sleep (*F*(1, 213) = 4.49, *p* = .035, *η*^2^ = 0.02), indicating a small effect (Figure 1)^2^.

Bonferroni-adjusted simple-effects analysis revealed a simple effect of insomnia, such that first responders with comorbid PTSD and insomnia reported mean fear of sleep scores (*M* = 9.65, *SD* = 8.53, *n* = 34) that were 6.17 points higher (*p* < .001, 95% confidence intervals [CI] = 3.11 to 9.23) than those with PTSD only (*M* = 3.44, *SD* = 4.66, *n* = 16). First responders with insomnia only (*M* = 4.21, *SD* = 6.48, *n* = 48) also reported mean fear of sleep scores that were 2.41 points higher (*p* = .007, 95% CI =.68 to 4.14) than participants with neither insomnia nor PTSD (*M* = 1.51, *SD* = 2.96, *n* = 121). There were no differences in fear of sleep scores between first responders with either PTSD only or insomnia only, as indicated by overlapping 95% CIs (Figure 1).

### Hyperarousal and trauma-related nightmares are independently associated with fear of sleep

Based on our ANCOVA model’s significant interaction, we conducted post hoc regression analyses to investigate which PTSD symptoms were most associated with fear of sleep. We entered each of the four PTSD symptom clusters (intrusions, avoidance, negative alterations in cognitions and mood, and hyperarousal) as predictors of fear of sleep. Because nightmares are one proposed cause of fear of sleep, we removed the trauma-related nightmare item from the PTSD intrusions cluster (PCL item 2) and entered this as a separate predictor. We also removed the insomnia item from the PTSD hyperarousal cluster (PCL item 20) to isolate the association between non-sleep-related alterations in arousal and reactivity (e.g. hypervigilance and exaggerated startle response) on fear of sleep. All PTSD symptoms were entered in the same step. We then entered insomnia status (ISI ≥ 15), sex, and years of service as covariates in a subsequent step (each trauma load variable was nonsignificant and excluded from these analyses as well). All beta weights represent standardized regression coefficients.

PTSD hyperarousal symptoms (*β* = .29, *SE* = .12, *p* = .001) and trauma-related nightmares (*β* = .22, *SE* = .47, *p* =.007) had the most robust associations with fear of sleep, even after adjusting for insomnia, sex, and years of service (Table 4). The full model including all predictors explained 33.7% of variance in fear of sleep (*R*^2^ = .337), indicating a medium effect [34]. Table 4 shows the results of our post hoc models.

**Table 4.** Post Hoc Multivariate Linear Analyses Regressing PTSD Symptom Clusters and Trauma-Related Nightmares on Fear of Sleep (*N* = 242)

	Unadjusted model					Adjusted model				
Predictor	*β* (SE)	*p*	95% CI	*R* ^2^	Adjusted *R*^2^	*β* (SE)	*p*	95% CI	*R* ^2^	Adjusted *R*^2^
Intrusions-S	0.04 (0.18)	.747	−0.30, 0.42	29.9%	28.3%	0.00 (0.19)	.985	−0.37, 0.38	33.7%	31.2%
Avoidance	−0.02 (0.25)	.867	−0.54, 0.46			0.00 (0.26)	1.000	−0.50, 0.51		
Cognitions and mood	0.11 (0.09)	.269	−0.08, 0.28			0.07 (0.09)	.454	−0.11, 0.25		
*Hyperarousal-S*	*0.29 (0.12)*	*.001*	*0.19, 0.67*			*0.25 (0.13)*	*.005*	*0.11, 0.62*		
*Trauma-related nightmares*	*0.22 (0.47)*	*.007*	*0.35, 2.19*			*0.20 (0.48)*	*.018*	*0.20, 2.12*		

Significant results are in italics.

Unadjusted model = model only included variables shown, entered in single step; Adjusted model *=* model included variables shown, entered in single step, while adjusting for insomnia status (Insomnia Severity Index score ≥15), sex, and years of service. *β* = standardized regression coefficient; SE = standard error; *p* = significance value; 95% CI = confidence intervals; *R*^2^ = portion of variation in fear of sleep explained by model (adjusted *R*^2^ corrects for the number of predictors in the model): small = 0.04, medium = 0.25, large = 0.64 (Ferguson, 2009). Intrusions-S = PTSD intrusions symptom cluster minus nightmare item (sum of PCL-5 items 1, 3, 4, and 5); Avoidance = PTSD avoidance symptom cluster (sum of PCL-5 items 6 and 7); Cognitions and Mood = Negative alterations in cognitions and mood PTSD symptom cluster (sum of PCL-5 items 8–14); Hyperarousal-S = PTSD symptom clusters minus insomnia item (sum of PCL-5 items 15–19); Trauma-related nightmares (PCL item 2 = “Repeated, disturbing dreams of the stressful experience?” scored 0 [not at all] to 4 [extremely]).

## Discussion

This cross-sectional study examined fear of sleep among active-duty first responders with high rates of trauma exposure, psychopathology, and sleep disturbances. Consistent with prior studies, fear of sleep was positively associated with cumulative lifetime trauma, including interpersonal trauma-type clusters. We also replicated associations between fear of sleep and more severe psychopathology and sleep disturbances and extended this literature with the first evidence that fear of sleep was correlated with more alcohol use problems. Finally, fear of sleep was most severe in first responders reporting provisional PTSD comorbid with insomnia, and this effect was most robustly associated with hyperarousal symptoms and trauma-related nightmares.

### Fear of sleep across trauma-type clusters

We replicated previous findings that fear of sleep is associated with lifetime trauma load [14, 35] and exposure to specific trauma-type clusters characterized by predominant death threat (e.g. sudden violent death) and victimization (e.g. sexual assault) [22]. Predominant death threat traumas may be associated with fear of sleep because they encompass life-threatening events (e.g. being shot) that might engender nocturnal hypervigilance to increase perceptions of safety (“e.g. I tried to stay as alert as I could while lying in bed”). Victimization trauma types that involve sexual violence might contribute to sleep fears because the bedroom environment becomes a cue associated with the trauma memory [15]. The positive correlation between fear of sleep and victimization traumas may also explain our finding that female responders reported more sleep fears than males. That is, this sex difference could be a potential byproduct of females being disproportionately victims of sexual trauma [36], which might increase their fear of the bedroom or sleep-related contexts. Indeed, this same-sex difference in fear of sleep has been observed in prior studies [14, 15], and we also found an association between female sex and experiencing more victimization-type traumas.

### Fear of sleep is most severe in first responders with comorbid PTSD and insomnia

In line with previous work in trauma-exposed community adults [14, 15], fear of sleep was significantly elevated in first responders with comorbid PTSD and insomnia, controlling for sex and years of service. One notion is that fear of sleep develops in response to PTSD symptoms (e.g. nightmares) that drive maladaptive safety behaviors (e.g. hypervigilance) that disrupt sleep and perpetuate insomnia [10–12]. Indeed, our post hoc analyses lend preliminary support to this potential pathway, as trauma-related nightmares and the PTSD hyperarousal symptom cluster (which includes hypervigilance) had the most robust associations with fear of sleep, above and beyond the remaining PTSD clusters, insomnia status, sex, and years of service. That said, it is also possible fear of sleep contributes to PTSD and is not exclusively a response to it.

Importantly, hyperarousal symptoms were associated with fear of sleep without the insomnia item included in this symptom cluster, calling attention to the potential role of the other hyperarousal symptoms in fear of sleep. For instance, fear of sleep may reflect an underlying fear of losing vigilance (“I tried to stay alert to any strange noises while going to sleep”) [37], and this might be especially salient in first responders who were trained to be vigilant. In addition, a fear of sleep may reflect an inability to inhibit fear in the presence of safety, which is a putative biomarker of PTSD [38]. Notably, none of the trauma load variables were significant covariates in these models, supporting previous evidence that fear of sleep may be a consequence of PTSD rather than trauma exposure alone [39]. Nonetheless, it is possible that fear of sleep develops in response to certain trauma types (e.g. victimization), leading to disturbed sleep in the acute aftermath of trauma that increases future risk for PTSD. We need prospective studies that collect data on fear of sleep shortly after trauma to these hypotheses.

### First responders may misuse alcohol to manage fear of sleep

We found novel evidence that more severe fear of sleep is associated with increased risk for alcohol use disorder. Because alcohol is commonly used to downregulate fear and anxiety [40], it is possible that first responders with excessive sleep fears turn to alcohol to self-medicate, to achieve sleep more readily, or both. By extension, alcohol misuse might be another safety behavior that first responders with a fear of sleep rely on to mitigate fear but that worsens their sleep. Indeed, increased alcohol use was also associated with more severe anxiety and stress symptoms in our study, further implying alcohol may have been used to self-medicate strong emotions and promote relaxation. We encourage future investigators to explore further the relationship between fear of sleep, alcohol, and other substance use.

### Early-career first responders have a greater fear of sleep

Our exploratory correlations indicated first responders with more years working in public safety reported less fear of sleep. Taken with the observation that years of service were also correlated with less severe PTSD and depression symptoms in our sample, this suggests a possible self-selection bias such that the individuals who persist in first responder occupations may be more resilient to traumatic stress. Previous studies on first responders have reported a similar link between more years of service and less severe psychopathology, albeit with mixed findings [41]. Spending more years in public safety might also mean more time for acquiring skills that enhance self-efficacy and coping, both of which could confer protective effects against a fear of sleep and other posttraumatic outcomes [41]; conversely, this may also suggest that first responders with less experience may be most vulnerable to developing sleep fears. Prospective studies could better delineate the development of fear of sleep across the careers of first responders and the myriad factors that may influence its course. Additionally, future work should assess duty-related trauma specifically to better address how first responder occupations explain the relationship between fear of sleep and relevant health outcomes.

### Fear of sleep is worse when sleeping alone

Our exploratory correlations also found that more nights per week of sleeping alone was associated with worse fear of sleep. This might suggest first responders are more fearful while sleeping alone, and that bed sharing may potentially serve to reduce sleep fears (and thus may be another possible safety behavior). Conversely, it could also be the case that responders with a fear of sleep choose to sleep in a different room, perhaps to avoid the bed [12], maintain vigilance [37], delay sleep, or avoid disturbing their partners with these behaviors. This interpretation comports with another study that found a moderate (albeit trending) association between more fear of sleep and less time in bed (*r* = −0.28, *p* = .07) as assessed via home-based polysomnography [12]. We need more prospective research on this relationship to fully appreciate its potential clinical and treatment implications.

## Clinical Implications

Given our finding that fear of sleep is associated with more severe clinical profiles, our study suggests active duty first responders with comorbid PTSD and insomnia may benefit from treatments that ameliorate fear of sleep. This may be especially relevant for female responders, new recruits, those exposed to high rates of interpersonal trauma, and those experiencing nightmares. For example, several studies have shown that a brief behavioral treatment for nightmares reduces fear of sleep [42, 43]. Preliminary evidence also suggests cognitive behavioral therapy for insomnia (CBT-I) attenuates fear of sleep, even without tailoring treatment to focus on sleep fears specifically [12]. Moreover, using CBT-I to reduce sleep fears prior to first-line PTSD interventions may improve treatment response [44]. One trial adapted CBT-I to address fear of sleep explicitly by focusing on sleep-maladaptive thoughts and behaviors gleaned from the Fear of Sleep Inventory, but fear of sleep was not a reported outcome [45]. Future research could test whether these or similar modifications produce more clinically meaningful reductions in sleep fears than standard CBT-I. Before doing so, however, it would be important for researchers to first identify cutoff scores for detecting the point(s) at which fear of sleep becomes clinically significant, as this remains unclear [13]. For example, levels of fear of sleep vary considerably across studies of trauma-exposed adults [14, 23] and adolescents [39, 46], and these discrepancies underscore the need to better understand when fear of sleep warrants intervention.

## Limitations and Future Directions

Our findings should be considered within the context of our study’s limitations. First, the cross-sectional design precludes our ability to infer directionality. For instance, although we hypothesize PTSD may drive fear of sleep, there is prospective evidence for both the inverse and a bidirectional relationship [47, 48]. Second, we operationalized trauma-related nightmares using a single item from the PCL-5. While this is frequently done to assess nightmares [49], this item does not assess whether participants were awoken by their dreams, which is often used to distinguish nightmares from bad dreams [50]. It is also important to measure idiopathic nightmares as well as their frequency, functional impairment, and duration, which can be assessed with the Nightmare Disorder Index or the Disturbing Dreams and Nightmare Severity Index. Third, due to researcher error, our measure of PTSD assessed symptoms in relation to a “stressful life event,” yet our intention was to query about “stressful work-related events” to capture duty-related trauma. This means we could have potentially measured PTSD symptoms in relation to less severe experiences that might not satisfy the criterion A definition of trauma, although it is conceivable this would have rarely occurred given the high rates of criterion A trauma exposure reported by our sample on the LEC-5. Further, because the LEC-5 did not query about duty-related trauma, participants may have reported traumatic events they experienced outside their first responder occupations, thus limiting our interpretation of how fear of sleep correlates with occupational trauma specifically. Fourth, we assessed PTSD symptoms from the past week only, thus we cannot say a PCL-5 score of ≥33 indicates probable PTSD because this requires symptoms to persist for at least one month. Future studies could better assess trauma and PTSD using a structured interview (e.g. the Clinician-Administered PTSD Scale for DSM-5). Fifth, 86.4% of our sample identified as white and 4.9% as Hispanic/Latino(a). Therefore, the findings of the current study may not generalize to first responders who are more racially or ethnically diverse. Sixth, it is possible fear of sleep is also associated with other non-prescribed substance use besides alcohol. Seventh, we did not assess for other sleep disorders (e.g. obstructive sleep apnea) or other aspects of sleep health (e.g. daytime sleepiness), the presence of which may have impacted our results. Eighth, data collection occurred during the COVID-19 pandemic, which may have affected the sleep health and functioning of our first responder sample. Despite these limitations, our study is focused on a novel, highly traumatized sample. We also utilized empirically derived trauma-type clusters, thereby building on previous attempts to assess differences in fear of sleep between *subjective* trauma-type classifications, which may have contributed to null findings in prior studies [12].

## Conclusions

Fear of sleep was most severe in first responders with provisional PTSD comorbid with insomnia and was associated with exposure to interpersonal trauma-type clusters, more severe psychopathology, alcohol use problems, and sleep disturbances. Our findings align with the conceptualization that sleep fears are associated with PTSD hyperarousal symptoms, trauma-related nightmares, and cooccurring insomnia and extend the clinical relevance of fear of sleep to active duty first responders. This population may benefit from treatments that lower fear of sleep to optimize sleep quality, mental health, and potentially work performance.

## Supplementary Material

zpad053_suppl_Supplementary_Tables_1

## Footnotes

1 We excluded one participant from trauma exposure analyses due to suspected underreporting (denied any lifetime trauma exposure despite serving as a law enforcement officer for ≥20 years).

2 Results did not differ substantively after removing the nightmare and insomnia items (PCL items 2 and 20) from the PTSD variable: (*F*(1, 213) = 6.81, *p* = .010, *η*^2^ = 0.03).
