# Supplementary material for: Fear of sleep in first responders: associations with trauma types, psychopathology, and sleep disturbances
Source: Sleep Adv. 2023 Dec 1;4(1):zpad053. doi: 10.1093/sleepadvances/zpad053 (PMC10718811; doi:10.1093/sleepadvances/zpad053)
Supplement: zpad053_suppl_Supplementary_Tables_1 [file zpad053_suppl_supplementary_tables_1.docx]

**Title:** Fear of sleep in first responders: Associations with trauma types, psychopathology, and sleep disturbances

**Authors:** Anthony N. Reffi, PhD^a^, David A. Kalmbach, PhD^a^, Philip Cheng, PhD^a^, Peter Tappenden, BA^b^, Jennifer Valentine, MA^b^, Christopher L. Drake, PhD^a^, Wilfred R. Pigeon, PhD^cd^, Scott M. Pickett, PhD^e^, Michelle M. Lilly, PhD^b^

^a^Thomas Roth Sleep Disorders & Research Center, Henry Ford Health System, Detroit, MI, USA*

^b^Department of Psychology, Northern Illinois University, DeKalb, IL, USA*

^c^VISN 2 Center of Excellence for Suicide Prevention, 400 Fort Hill Ave., Canandaigua, New York 14424, USA

^d^University of Rochester Medical Center, 300 Crittenden Blvd. – Box PSYCH, Rochester, NY 14642, USA

^e^Center for Translational Behavioral Science, Florida State University College of Medicine, Tallahassee, Florida, USA

*This research was conducted at the Sleep Disorders and Research Center at Henry Ford Health System, Detroit, MI, and Northern Illinois University, DeKalb, IL.

**Correspondence:** Anthony N. Reffi

Thomas Roth Sleep Disorders & Research Center

Department of Sleep Medicine

Henry Ford Health System

39450 West Twelve Mile Road

Novi, Michigan 48377

**Tel:** (313) 805-3080

**Email:** [areffi1@hfhs.org](mailto:areffi1@hfhs.org)

| **Supplemental Table 1** Independent samples t-tests comparing fear of sleep based on demographics and clinically significant mental health symptoms, hazardous alcohol use, and sleep disturbances (*N* = 242) | | | | | | | |
| --- | --- | --- | --- | --- | --- | --- | --- |
| Variable | Groups | *n* | *M* | *SD* | *t* | *p* | *g* |
| Sex | Male | 131 | 2.63 | 4.92 | **-2.53**^a^ | **.013** | **0.37** |
|  | Female | 91 | 4.75 | 6.89 |  |  |  |
| Occupation | Law enforcement officer | 135 | 3.46 | 5.51 | -0.82 | .412 | 0.12 |
|  | 9-1-1 telecommunicator | 72 | 4.19 | 7.16 |  |  |  |
| Work schedule | Night shift work | 130 | 4.09 | 6.55 | -1.52 | .129 | 0.22 |
|  | Standard schedule | 74 | 2.76 | 4.94 |  |  |  |
| Mental health | PTSD | 51 | 7.98 | 8.07 | **-4.88**^a^ | **< .001** | **1.06** |
|  | No PTSD | 177 | 2.24 | 4.34 |  |  |  |
|  | Depression | 65 | 6.94 | 8.41 | **-4.40**^a^ | **< .001** | **0.87** |
|  | Mild depression | 163 | 2.17 | 3.77 |  |  |  |
|  | Anxiety | 80 | 6.79 | 7.88 | **-5.44**^a^ | **< .001** | **0.93** |
|  | Mild anxiety | 148 | 1.76 | 3.37 |  |  |  |
|  | Stress | 55 | 7.35 | 8.66 | **-4.17**^a^ | **< .001** | **0.92** |
|  | Mild stress | 173 | 2.31 | 4.01 |  |  |  |
| Alcohol use | Hazardous – harmful | 41 | 5.27 | 6.32 | -1.98^a^ | .053 | 0.36 |
|  | Abstinent – low risk | 187 | 3.14 | 5.74 |  |  |  |
| Sleep disturbances | Trauma-related nightmares | 54 | 7.24 | 8.22 | **-4.17**^a^ | **< .001** | **0.88** |
|  | No – mild nightmares | 174 | 2.37 | 4.38 |  |  |  |
|  | Insomnia | 84 | 6.61 | 7.87 | **-5.41**^a^ | **< .001** | **0.90** |
|  | No insomnia | 143 | 1.74 | 3.22 |  |  |  |
| *Note*. Significant differences are bolded (*p* < .05).  Night shift work = regularly worked outside the hours of 0700 – 1800 over the past month; PTSD = Posttraumatic Stress Disorder Scale for DSM-5 (PCL-5) sum score ≥ 33, indicating provisional PTSD over the past week; depression, anxiety, and stress = Depression Anxiety and Stress Scale (DASS-21) sum scores ≥ 14, 10, or 19, respectively, indicating at least moderate severity for each symptom subscale over the past week; hazardous or harmful alcohol consumption = Alcohol Use Disorders Identification Test sum score ≥ 8; trauma-related nightmares = clinically significant distress over trauma-related nightmares assessed for the past week using the PCL-5 item 2 (“Repeated, disturbing dreams of the stressful experience”) score ≥ 2 (moderate distress); insomnia = Insomnia Severity Index sum score ≥ 15, indicating provisional insomnia over the past two weeks.  *n* = group size; *M* = mean; *SD* = standard deviation; *t* = t-statistic; *p* = significance value; *g* = Hedges’ *g* effect size: small = 0.2, medium = 0.5, large = 0.8.  ^a^Equal variances not assumed. | | | | | | | |
